# Reverse Knot Technique for Face Lifting With Polydioxanone Thread

**DOI:** 10.1111/jocd.70878

**Published:** 2026-05-05

**Authors:** Ana Paula Dornelles Manzoni, Alessandra Haddad, Andréia Fogaça, Valeska Ronsoni

**Affiliations:** ^1^ Complexo Hospitalar Santa Casa de Porto Alegre Universidade Federal de Ciências da Saúde de Porto Alegre Porto Alegre Rio Grande do Sul Brazil; ^2^ Universidade Federal de São Paulo e Hospital Israelita Albert Einstein São Paulo São Paulo Brazil; ^3^ Instituto Andréia Fogaça São Paulo São Paulo Brazil

**Keywords:** facial asymmetry, heavy face, nonsurgical lift, polydioxanone, thread, thread‐lift

## Abstract

**Background:**

The use of polydioxanone thread has grown substantially for facial soft‐tissue repositioning and collagen biostimulation, with a well‐established safety profile and favorable tissue integration.

**Aims:**

To describe the reverse knot technique, a novel method for the implantation of polydioxanone suspension threads.

**Patients/Methods:**

We present an original polydioxanone thread‐lifting technique designed primarily for patients with a “heavy face.” The reverse knot technique positions the knot in the anterior portion of the face, below the zygomatic cutaneous ligament, whereas anchoring the distal end of the thread in the temporal fascia, creating a lever mechanism that enhances lifting of the middle and lower thirds of the face.

**Conclusions:**

The technique provides immediate and effective midface soft‐tissue support, representing a minimally invasive alternative for face lifting and resulting in high patient satisfaction and minimal morbidity. The procedure should be performed by professionals experienced in thread lifting, as positioning the knot in the medial portion of the face increases support but also raises the risk of localized depressions at the knot site.

## 1 Introduction

The use of polydioxanone thread has grown substantially for facial soft‐tissue repositioning and collagen biostimulation. This type of thread is considered safe and capable of predictable tissue integration [1, 2]. Major challenges lie in refining application techniques and defining appropriate indications to achieve reproducible, durable, and safe outcomes. This article describes the reverse knot technique, a novel method for placing barbed polydioxanone suspension threads with a caudal‐to‐cranial vector, anchored by a knot positioned in the medial portion of the face. Unlike conventional techniques in which fixation occurs posteriorly, the reverse knot technique maximizes lifting effect and prolongs mechanical support through an anterior fixation point. The procedure should be performed by professionals with experience in thread lifting, as positioning the knot in the medial portion of the face increases lifting support but also raises the risk of localized depressions at the knot site.

## 2 Methods

This was an observational, descriptive, uncontrolled study reporting clinical experience with a novel technique for improving facial contour, especially in patients with a “heavy face.”

### 2.1 Patient Selection

Patients with a “heavy face,” defined as abundant superficial fat compartments predominantly in the middle and lower thirds of the face, resulting in a wider or poorly defined lower facial contour, were considered eligible. Exclusion criteria were pregnancy or breastfeeding, uncontrolled systemic disease, immunosuppression, risk of keloid formation, unrealistic aesthetic expectations, or cases in which surgical correction was indicated.

### 2.2 Thread Characteristics

Polydioxanone suspension threads with molding cogs (19G, 100 × 185 mm, United States Pharmacopeia [USP] 3.0, bidirectional) were used.

### 2.3 Reverse Knot Technique

**FIGURE 1:**
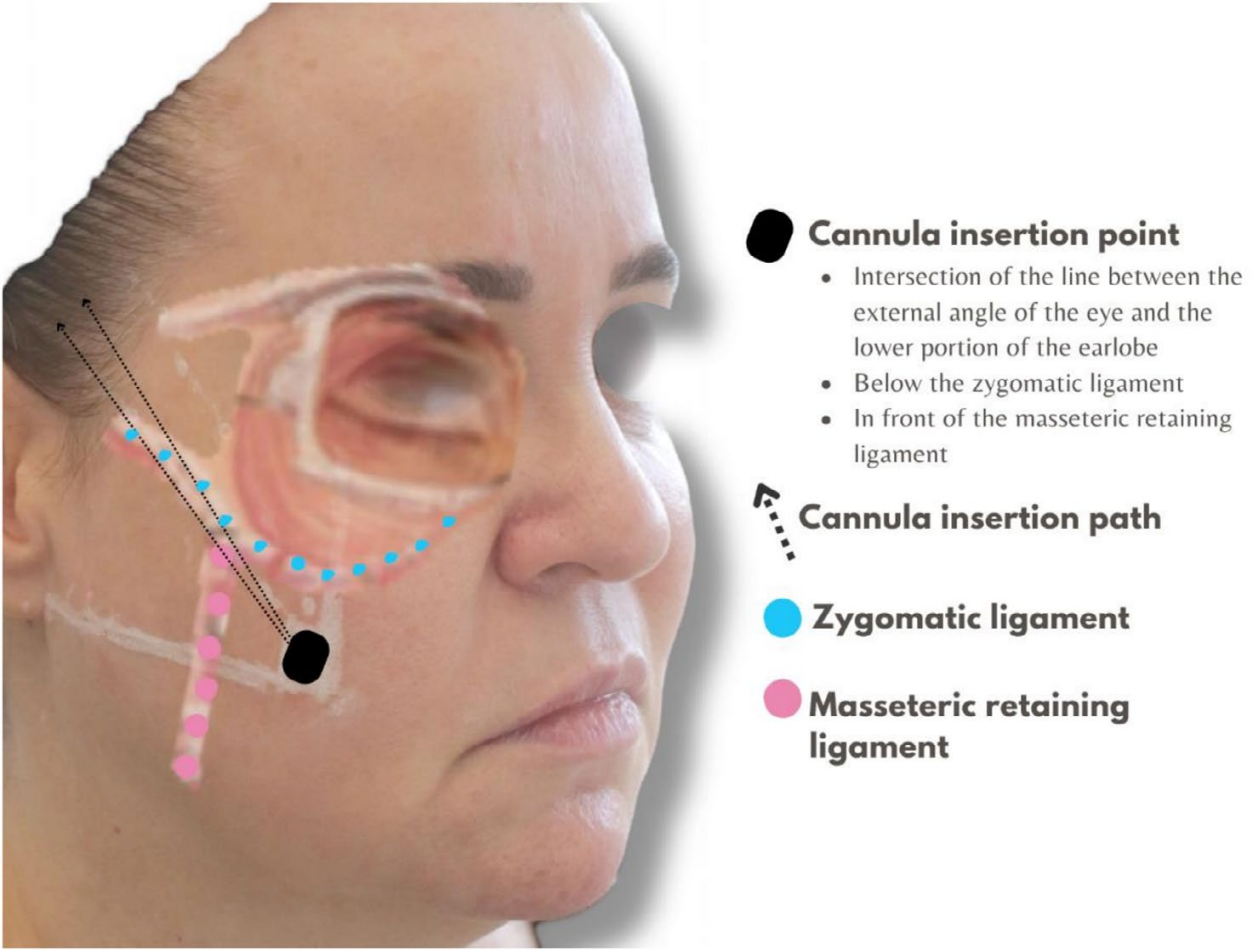
Reference points for applying the reverse knot technique.

The reverse knot technique (see Video 1 and Figure 1) is performed as follows:

- Antisepsis: The face and posterior temporal region, including hair and scalp, are cleaned with 2% alcoholic chlorhexidine, and a sterile field is established.
- Entry point: The entry point is located at the intersection of straight vertical and horizontal lines drawn from the lower portion of the earlobe and the lateral outer canthus. This point is positioned below the zygomatic cutaneous ligament and anterior to the masseteric retaining ligament.
- Cannula trajectory: From the entry point, the cannula is advanced toward the posterior temporal region, approximately 1 cm above the helix of the ipsilateral ear.
- Anesthesia: Boluses of 0.2 mL of 2% lidocaine with vasoconstrictor are administered at approximately 2‐cm intervals along the cannula trajectory.
- Entry port: A 16G needle is inserted perpendicular to the skin until reaching the middle‐to‐deep portion of the superficial fat compartments.
- Thread introduction: Two threads are introduced individually and in parallel. The cannula is advanced through the superficial fat compartments, and the distal portion of each thread is anchored approximately 1 cm beyond the posterior temporal region.
- Knot formation: The first knot is tied under sufficient traction to elevate the middle third of the face without causing skin puckering. It must be completely buried in the subcutaneous tissue, ensuring absence of skin dimpling or depression. The second knot is advanced toward the first and fixed at the depth of the superficial subcutaneous fat pad. It must also be completely buried in the subcutaneous tissue, ensuring absence of skin dimpling or depression.
- Thread trimming: Excess thread is trimmed close to the knot using sterile scissors.

**VIDEO 1:** Demonstration of the reverse knot technique for facial lifting with polydioxanone threads. Video content can be viewed at https://onlinelibrary.wiley.com/doi/10.1111/jocd.70878.

## 3 Results

Over a 4‐year period, 48 patients were treated with the reverse knot technique. In all cases, immediate and visible improvement in midface volume and mandibular contour was observed, with effective lifting of the lower third and high patient satisfaction. Complications included localized pain, edema, and ecchymosis, all of which were transient and typically occurred after thread placement. The only complication requiring intervention was skin dimpling at the cannula insertion site, which occurred in 9 patients and was fully corrected using subcision with a 21G needle. No cases of thread migration or facial asymmetry were observed.

**FIGURE 2:**
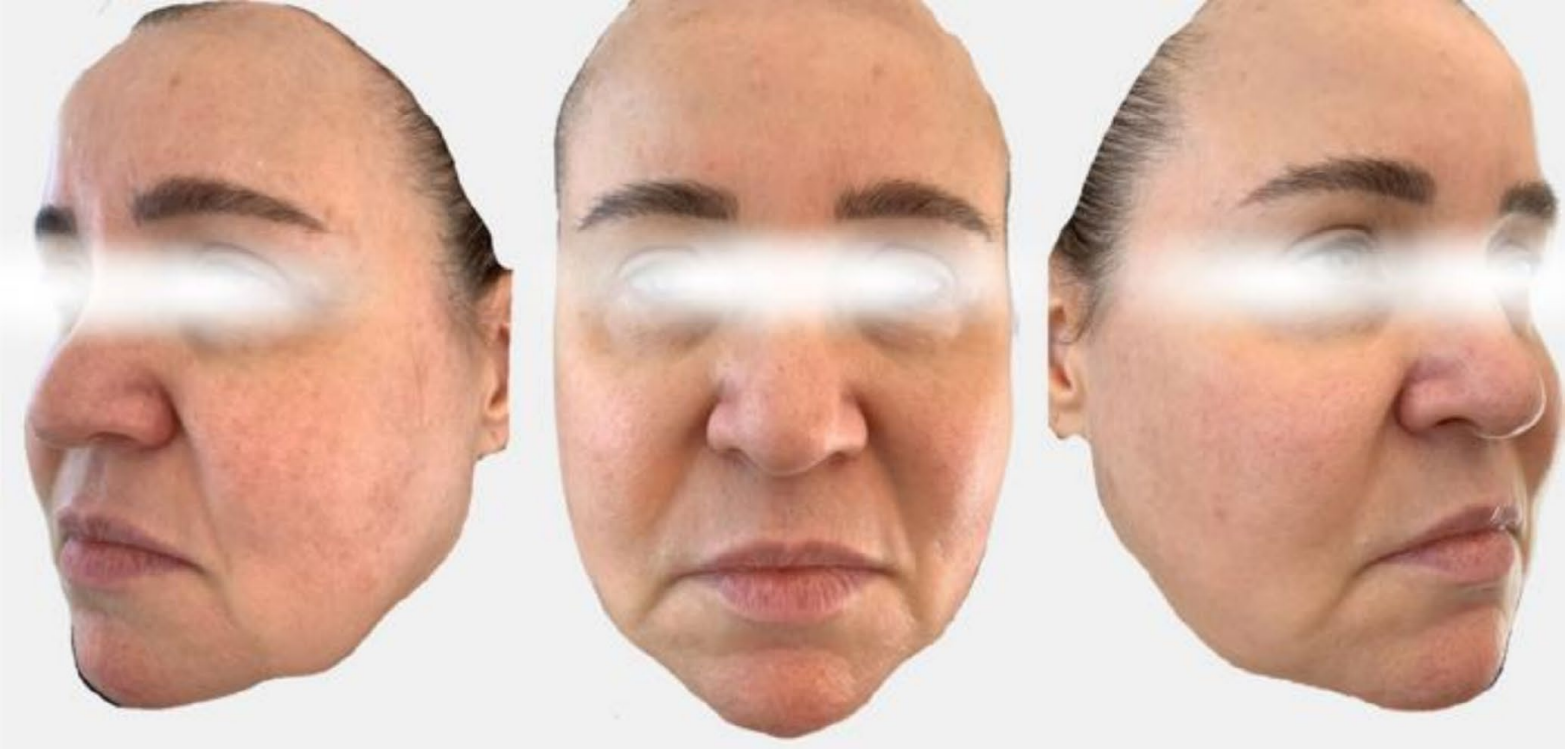
Pre‐treatment—57‐year‐old patient with volumetric displacement of subcutaneous tissue toward the middle and lower thirds of the face.

**FIGURE 3:**
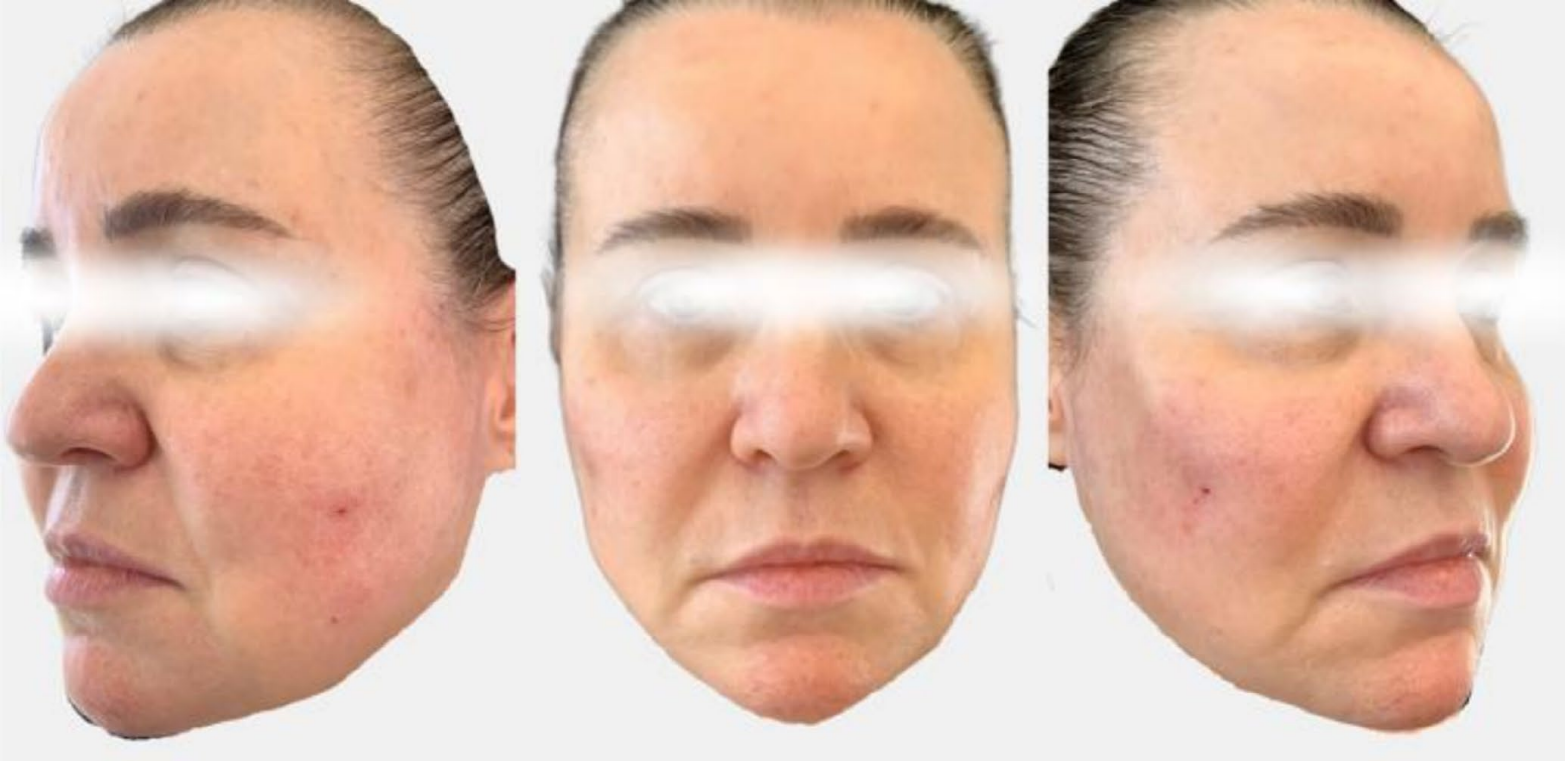
Immediate post‐treatment with the reverse knot technique—repositioning of the middle third and improvement of the mandibular contour through caudal‐to‐cranial elevation of the facial volume.

The patient illustrated in Figures 2 and 3 was 57 years of age and presented with a “heavy face.” Two polydioxanone suspension threads were placed per hemiface, resulting in repositioning of the middle and lower thirds and improved mandibular contour (Figures 2 and 3). In contrast to posterior knot techniques, which often show partial loss of mechanical traction within the first month, the reverse knot technique maintained mechanical traction for at least 3 months.

## 4 Discussion

Polydioxanone has been used in synthetic absorbable sutures for more than 40 years and is recognized for its safety, biocompatibility, and predictable tissue integration [1, 3]. The use of polydioxanone thread for facial suspension and collagen biostimulation offers a minimally invasive alternative to surgical lifting [4].

Despite the development of multiple thread‐lifting techniques to individualize treatment, achieving effective and long‐lasting tissue support remains challenging, especially in patients with a “heavy face” [5, 6]. Anatomical findings on the retaining ligaments of the face suggest that repositioning the zygomatic cutaneous ligament plays a key role in elevating the middle and lower thirds of the face [7, 8]. In the reverse knot technique, fixation below the zygomatic cutaneous ligament and anterior to the masseteric retaining ligament creates a lever effect that directs traction from the middle third of the face toward the temporal region, where the distal point of the thread is fixed.

The primary challenge of this technique lies in accurate thread placement and complete burial of the knot, since superficial positioning increases the risk of skin dimpling, causing depressions in the anterior portion of the middle third of the face. Risk mitigation includes the use of a 16G entry needle and molding cogs (which provide a smoother surface than knots made with barbed thread), limiting thread thickness to USP 3.0, and selecting patients with sufficient superficial malar fat compartments to allow the knot to be positioned deeply. Since adopting these technical specifications, no additional cases of localized depression at the knot site have occurred.

We recommend that this technique be reserved for professionals with advanced experience in thread lifting, as positioning the fixation knot in the medial portion of the face enhances lifting support but also increases the risk of localized depressions at the knot site. For this reason, meticulous technique and precise placement are crucial to minimizing complications. Particular attention must be given to maintaining the cannula trajectory through the superficial subcutaneous plane of the anterior face (which is characteristically more abundant), and subsequently, of the posterior face (which is significantly thinner). Inadequate correction of the plane during cannula advancement may damage the superficial temporal artery and the zygomatic and temporal branches of the facial nerve [9]. Although the reverse knot technique provides effective facial support and repositioning, we suggest complementing it with the placement of at least 2 additional suspension threads in the lower third of each hemiface to optimize outcomes and ensure traction durability.

This study has some limitations. First, the absence of a control group for comparison with conventional thread lifting techniques limits the objective assessment of the superiority of the technique. Second, the follow‐up period was limited to 3 months, and long‐term outcomes remain to be determined. Finally, outcome assessment relied on clinical examination and photographic comparison, without the use of standardized measurement tools. Additional controlled studies are therefore needed to confirm these findings and to validate the long‐term efficacy of the reverse knot technique. Since the purpose of this publication is to introduce a novel technique, further investigations involving comparative designs and larger samples are warranted.

## 5 Conclusions

The reverse knot technique using polydioxanone suspension thread is a minimally invasive aesthetic procedure that provides immediate and effective tissue support to the middle and lower thirds of the face. It may be particularly suitable for patients with a “heavy face,” since the traction applied to the support thread is more effectively stabilized by the knot positioned in the mobile face.

## Author Contributions

Dr. Ana Paula Dornelles Manzoni (corresponding author) developed the technique and performed the procedure with the assistance of Dr. Valeska Ronsoni. The other authors contributed to the literature review and the writing of the manuscript. All authors reviewed and approved the final version of the manuscript and agreed to be accountable for all aspects of the work.

## Funding

This research did not receive any specific grant from funding agencies in the public, commercial, or not‐for‐profit sectors.

## Ethics Statement

The study was conducted in accordance with the ethical principles of the Declaration of Helsinki. The patient provided written informed consent for the procedure and for the use of her images for academic and scientific purposes.

## Consent

Informed consent was obtained from all patients included in this study. The protocol follows the principles of the Declaration of Helsinki.

## Conflicts of Interest

The authors declare no conflicts of interest.

## Data Availability

The data that support the findings of this study are available from the corresponding author upon reasonable request.
